# A Treatise on Surgery by American Authors

**Published:** 1902-01

**Authors:** 


A Treatise on Surgery by American Authors. For Students and Practitioners of Medicine and Surgery. Edited by Roswell Park, M. D., Professor of Surgery in the University of Buffalo, N. Y. New (3d) edition in one Royal Octavo volume of 1350 pages with 692 engravings and 64 full-page plates in colors and monochrome. Cloth, $7.00 net; leather, $8.00 net. Lea Brothers & Co., Philadelphia and New York.

As might be expected its 1350 pages make this volume somewhat heavy and unwieldly. However, this fault is more than offset by the very positive advantage of having the entire work in one comprehensive volume. Thus are served the ends of both economy and convenience.

The subject matter is arranged in six main divisions as follows: I. Surgical Pathology. II. Surgical Diseases. III. Surgical Principles and Methods and Minor Proceedings. IV. Injury and Repair. V. Surgical Affections of the Tissues and Tissue Systems. VI. Special and Regional Surgery.

Parts I and II, comprising 220 pages, are written almost entirely by Dr. Park himself; Part III, by Drs. John Parmeter and H. A. Hare; Part IV, by Dr. Chas. B. Nancrede, of the University of Michigan; Parts V and VI, covering about 1000 pages, have as contributors a number of distinguished authors, including Drs. Park, H. H. Mudd, Duncan Eve, Chas. Stedman Bull, M. H. Richardson, Rudolph Matas of Tulane, and others.

The work deserves the highest praise. It is excellent in every particular. The present edition has been enriched by the addition of many new illustrations. There are now 64 full-page colored plates and nearly 700 engravings. R.

